# Integrative Analyses of mRNA Expression Profile Reveal the Involvement of *IGF2BP1* in Chicken Adipogenesis

**DOI:** 10.3390/ijms20122923

**Published:** 2019-06-14

**Authors:** Jiahui Chen, Xueyi Ren, Limin Li, Shiyi Lu, Tian Chen, Liangtian Tan, Manqing Liu, Qingbin Luo, Shaodong Liang, Qinghua Nie, Xiquan Zhang, Wen Luo

**Affiliations:** 1 Department of Animal Genetics, Breeding and Reproduction, College of Animal Science, South China Agricultural University, Guangzhou 510642, China; Jiahui_CHEN@stu.scau.edu.cn (J.C.); xueyi_ren@stu.scau.edu.cn (X.R.); lilimin@stu.scau.edu.cn (L.L.); lushiyi@stu.scau.edu.cn (S.L.); tian_cheng@stu.scau.edu.cn (T.C.); liangtian_tan@stu.scau.edu.cn (L.T.); liumanqing@scau.edu.cn (M.L.); qbluo@scau.edu.cn (Q.L.); sdliang@scau.edu.cn (S.L.); nqinghua@scau.edu.cn (Q.N.); xqzhang@scau.edu.cn (X.Z.); 2 Guangdong Provincial Key Lab of Agro-Animal Genomics and Molecular Breeding, South China Agricultural University, Guangzhou 510642, China; 3 Key Lab of Chicken Genetics, Breeding and Reproduction, Ministry of Agriculture, South China Agricultural University, Guangzhou 510642, China

**Keywords:** Chinese native broiler, abdominal fat deposition, adipogenesis, *IGF2BP1*, high-fat diet, differentially expressed gene

## Abstract

Excessive abdominal fat deposition is an issue with general concern in broiler production, especially for Chinese native chicken breeds. A high-fat diet (HFD) can induce body weight gained and excessive fat deposition, and genes and pathways participate in fat metabolism and adipogenesis would be influenced by HFD. In order to reveal the main genes and pathways involved in chicken abdominal fat deposition, we used HFD and normal diet (ND) to feed a Chinese native chicken breed, respectively. Results showed that HFD can increase abdominal fat deposition and induce adipocyte hypertrophy. Additionally, we used RNA-sequencing to identify the differentially expressed genes (DEGs) between HFD and ND chickens in liver and abdominal fat. By analyzed these DEGs, we found that the many DEGs were enriched in fat metabolism related pathways, such as peroxisome proliferator-activated receptor (PPAR) signaling, fat digestion and absorption, extracellular matrix (ECM)-receptor interaction, and steroid hormone biosynthesis. Notably, the expression of *insulin-like growth factor II mRNA binding protein 1 (IGF2BP1)*, which is a binding protein of *IGF2* mRNA, was found to be induced in liver and abdominal fat by HFD. Ectopic expression of *IGF2BP1* in chicken liver-related cell line Leghorn strain M chicken hepatoma (LMH) cell revealed that *IGF2BP1* can regulate the expression of genes associated with fatty acid metabolism. In chicken preadipocytes (ICP cell line), we found that *IGF2BP1* can promote adipocyte proliferation and differentiation, and the lipid droplet content would be increased by overexpression of *IGF2BP1*. Taken together, this study provides new insights into understanding the genes and pathways involved in abdominal fat deposition of Chinese native broiler, and *IGF2BP1* is an important candidate gene for the study of fat metabolism and adipogenesis in chicken.

## 1. Introduction

Chicken meat is one of the most consumed meats around the world, and the fast-growing commercial broiler chickens provide the most meat products than the other broiler breeds. However, consumers often prefer native broiler chickens over fast-growing breeds in China, as they are more suitable for Chinese eating habits [1]. In general, Chinese native broilers not only have slower growth-rate but also have excessive fat deposition. Proper quantities of subcutaneous fat and intramuscular fat are necessary for improving meat quality and flavor, but a higher abdominal fat content not only reduces feed efficiency and carcass yield, but also causes environmental pollution and rejection of the meat by the consumers [2,3]. Therefore, excess abdominal fat has been one of the main problems in broiler industry, especially in yellow feathered broiler chickens.

Insulin-like growth factor 2 mRNA-binding protein 1 (IGF2BP1), which can bind *IGF2* mRNA and affect its RNA target’s fate, belongs to a conserved family of single-stranded RNA-binding proteins [4,5]. It has been showed that *IGF2BP1* plays important roles in various aspects of cell function, such as cell proliferation, differentiation, migration, morphology and metabolism [5,6,7,8]. *IGF2BP1* regulates these cell functions by binding to its target RNAs and affecting their translatability, stability, or localization [9,10,11]. The main *IGF2BP1* target RNAs include *IGF2*, *c-Myc*, *CD44* and *Gli1* [12]. Recently, it has been found that IGF2BP1 is a *N*^6^-methyladenosine (m^6^A) readers and might bind with > 3000 mRNA transcript targets [13]. This profound role of *IGF2BP1* highlights its functional importance in post-transcriptional gene regulation. In addition, IGF2BP1 can play roles in a miRNA-dependent manner. It is able to binds the *SRF* mRNA and promotes *SRF* expression in several cancer cell lines by impairing the miRNA-dependent decay of the *SRF* mRNA [14]. However, even though the *IGF2BP1* plays an essential role in many cell processes, its function in fat deposition and adipogenesis still remain unclear. 

In this study, we analyzed the gene expression profiles of liver and abdominal fat from the chickens fed with a high-fat diet (HFD) or normal diet (ND), and found that *IGF2BP1* was differentially expressed in the two groups. *IGF2BP1* ectopic expression in chicken liver-related cell affected the expression of genes related to fat deposition and PPARγ signaling pathway. In chicken preadipocytes, *IGF2BP1* promoted cell proliferation and induced fat deposition related genes’ expression. Additionally, *IGF2BP1* can also induce adipocyte differentiation and increase lipid droplet accumulation. The findings of this study would be important for understanding the genes and pathways involved in chicken abdominal fat deposition, and it is also beneficial to understand the function of *IGF2BP1* in adipogenesis.

## 2. Results

### 2.1. High-Fat Diet Promotes Chicken Abdominal Fat Deposition and Induces Adipocyte Hypertrophy

**Figure 1:**
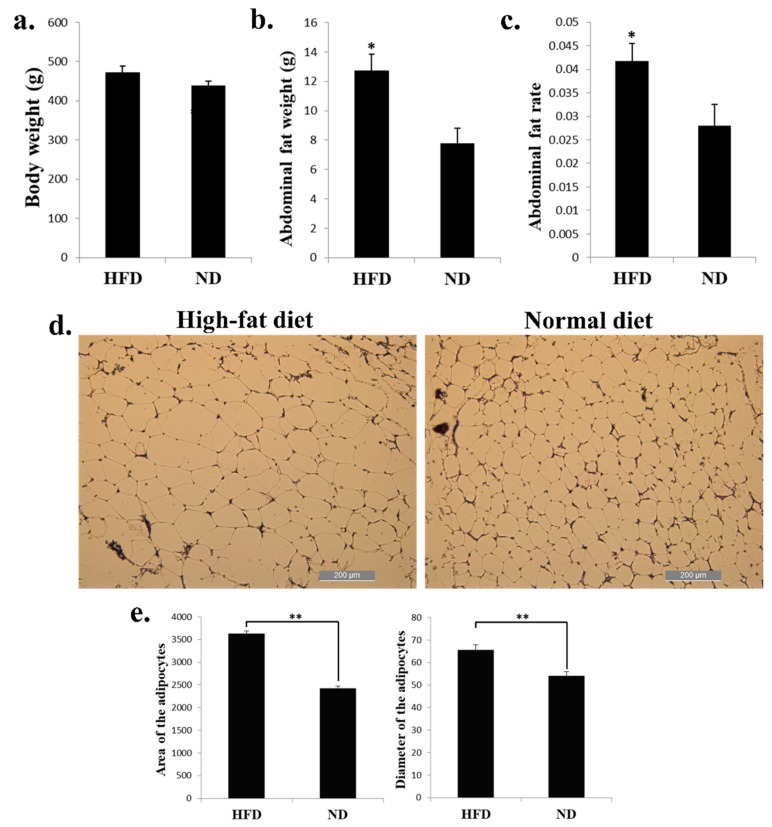
High-fat diet promotes chicken abdominal fat deposition and induces adipocyte hypertrophy. (**a**) Body weight of the broilers fed with high-fat diet (HFD) and normal diet (ND). (**b**) Abdominal fat weight of the broilers fed with HFD and ND. (**c**) Abdominal fat rate of the broilers fed with HFD and ND. (**d**) Micrograph of abdominal fat cross-section from HFD (left) and ND (right) chickens. Bar, 200 µm. (**e**) Area (left) and diameter (right) of the adipocyte from HFD and ND chicken abdominal fat. * *p* < 0.05; ** *p* < 0.01.

HFD can induce the expression of genes involved in adipogenesis and fat deposition. To find the genes and pathways related to chicken abdominal fat deposition, we fed the 8-week-old chickens with HFD and ND for 2 weeks, and the livers and abdominal fats were collected for further analysis. For the phenotypic changes of the chickens, the body weight of chickens fed with HFD have no significant change than the chickens fed with ND (Figure 1a). However, the abdominal fat weight and abdominal fat rate were significantly increased in HFD chickens than in ND chickens (Figure 1b,c). The adipocytes of the HFD chicken abdominal fat looks bigger than the ND group (Figure 1d). After calculated the area and diameter of the two groups’ adipocytes, we found that the HFD chickens have larger adipocytes area and longer adipocytes diameter than in ND chickens (Figure 1e). Therefore, these results demonstrated that a high-fat diet can promote chicken abdominal fat deposition and induces adipocyte hypertrophy.

### 2.2. Differentially Expressed Genes between HFD and ND Chickens

**Figure 2:**
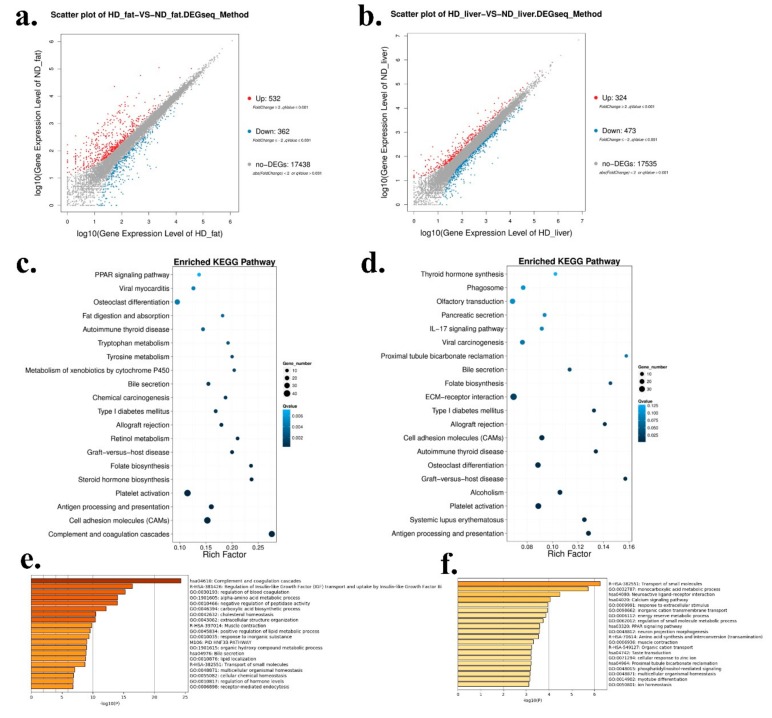
Differentially expressed genes between HFD and ND chickens. (**a**) Scatter plot of differentially expressed genes (DEGs) between HFD and ND chickens in abdominal fat. (**b**) Scatter plot of DEGs between HFD and ND chickens in liver. (**c**) Enriched Kyoto Encyclopedia of Genes and Genomes (KEGG) pathway of DEGs between HFD and ND chickens in abdominal fat. (**d**) Enriched KEGG pathway of DEGs between HFD and ND chickens in liver. (**e**) Gene Ontology (GO) enrichment of DEGs between HFD and ND chickens in abdominal fat. (**f**) GO enrichment of DEGs between HFD and ND chickens in liver.

As the liver and abdominal fat were the main tissues for fat synthesis and adipogenesis, we used these two tissues collected from HFD and ND chickens for RNA sequencing in order to find some genes and pathways involved in chicken abdominal fat deposition. In abdominal fat, a total of 532 genes were up-regulated and 362 genes were down-regulated in HFD chickens (Figure 2a and Supplementary File 1). In liver, a total of 324 genes were up-regulated and 473 genes were down-regulated in HFD chickens (Figure 2b and Supplementary File 2). Next, Kyoto Encyclopedia of Genes and Genomes (KEGG) pathway analysis was carried out to find the enriched pathways of the differentially expressed genes (DEGs). In abdominal fat, the enriched pathways included many fat deposition related pathways, such as PPAR signaling pathway, fat digestion and absorption, and steroid hormone biosynthesis (Figure 2c). However, the pathways in livers were mainly enriched in ECM-receptor interaction and Type I diabetes mellitus (Figure 2d). Furthermore, GO Enrichment Analysis was performed to analyze the functions of these DEGs. In abdominal fat, the DEGs’ functions were related to lipid localization and many metabolic processes, such as positive regulation of lipid metabolic process, alpha-amino acid metabolic process, and organic hydroxyl compound metabolic process (Figure 2e). In liver, the functions of the DEGs were related to the PPAR signaling pathway and the energy reserve metabolic process (Figure 2f), which are implicated in fat deposition.

### 2.3. The Expression of IGF2BP1 can be Significantly Induced in High-Fat Condition

**Figure 3:**
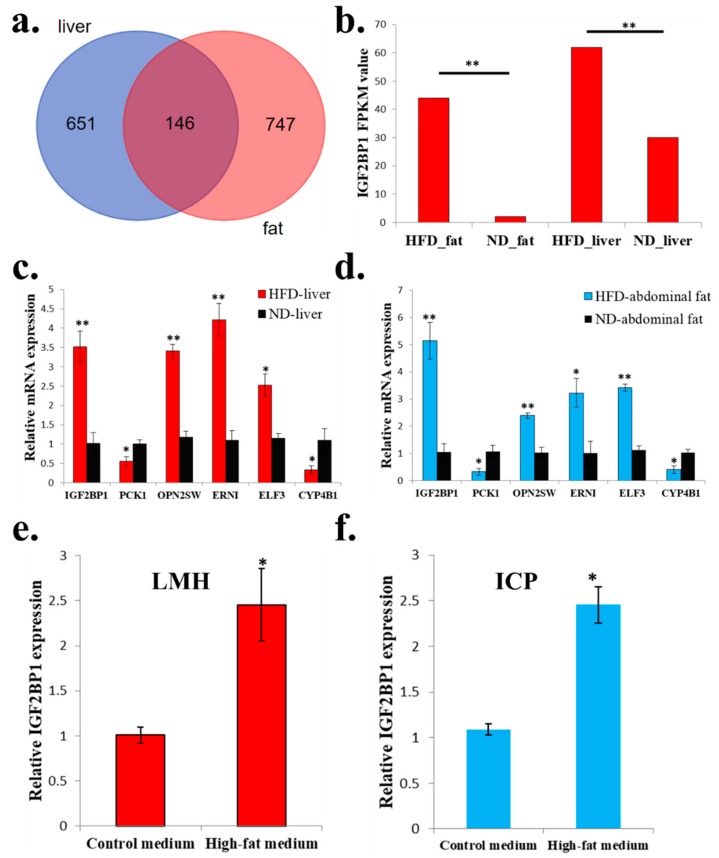
The expression of *IGF2BP1* can be significantly induced in a high-fat condition. (**a**) Specific and common DEGs in liver and abdominal fat. The three groups of the Venn diagram represent the liver specific DEGs between HFD and ND chicken (blue), common DEGs between HFD and ND chicken in liver and abdominal fat (deep red), and abdominal fat specific DEGs between HFD and ND chicken (light red), respectively. (**b**) RNA-sequencing revealed that HFD can significantly induce *IGF2BP1* expression in liver and abdominal fat of chicken. (**c**) qPCR validation of six DEGs obtained from RNA-seq in liver. (**d**) qPCR validation of 6 DEGs obtained from RNA-seq in abdominal fat. (**e**). High-fat medium can significantly promote *IGF2BP1* expression in chicken LMH cell. (**f**) High-fat medium can significantly promote *IGF2BP1* expression in chicken ICP cell. The data are mean ± S.E.M. with four samples (Figure 3b has three samples). Independent sample *t*-test was used to analyze the statistical differences between groups. * *p* < 0.05; ** *p* < 0.01.

Next, we want to find genes which are not only differentially expressed in abdominal fats, but also differentially expressed in livers between HFD and ND chickens. We found a total of 146 genes were suitable for this condition (Figure 3a and Supplementary File 3). Among these 146 genes, *IGF2BP1*, which is an *IGF2* mRNA binding protein and play important role in many cellular processes, was differentially expressed between HFD chickens and ND chickens (Figure 3b). To exam the quality and accuracy of RNA-seq, we used qPCR to validate the expression of six random DEGs that were among the 146 genes. Results confirmed the qPCR results were consistent with the RNA-seq results (Figure 3c,d). Finally, to verify whether high-fat condition can also induce *IGF2BP1* expression at the cellular level, we used high-fat medium and normal medium (control group) to culture chicken LMH cell and ICP cell. After 48 h of culture, *IGF2BP1* expression can be significantly induced by high-fat medium in both cell lines (Figure 3e,f). Therefore, these results suggest that the expression of *IGF2BP1* can be induced in a high-fat condition. 

### 2.4. IGF2BP1 Promotes the Expression of Genes Involved in Fatty Acid Metabolism in LMH Cell

**Figure 4:**
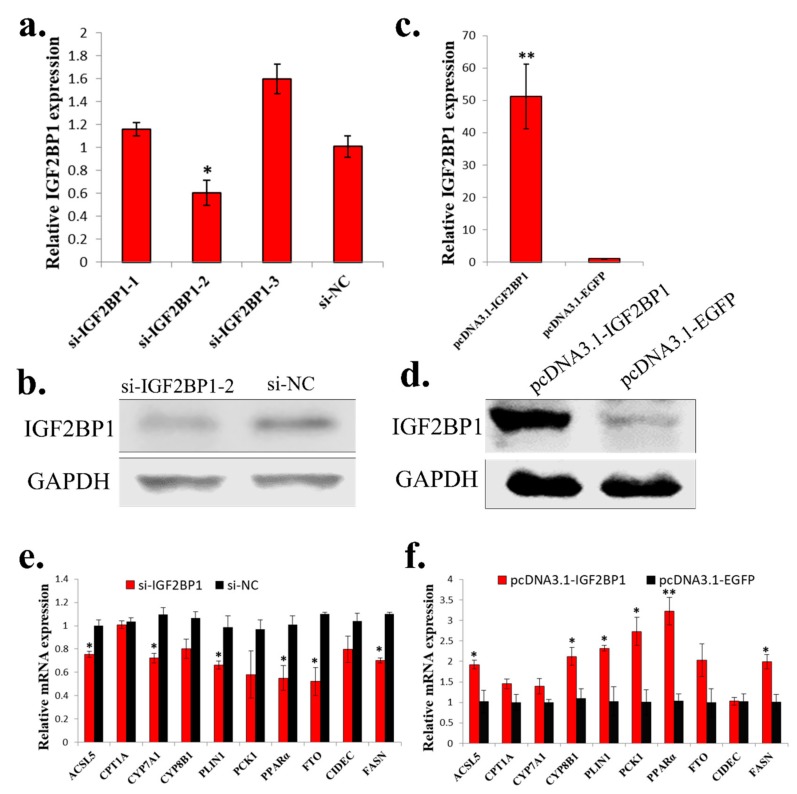
*IGF2BP1* promotes the expression of genes involved in fatty acid metabolism in LMH cell. (**a**) The mRNA expression of *IGF2BP1* after 48 h transfection of si-IGF2BP1 in LMH cell. (**b**) The protein expression of *IGF2BP1* after 48 h transfection of si-IGF2BP1 in LMH cell. (**c**) The mRNA expression of *IGF2BP1* after 48 h transfection of *IGF2BP1* overexpression vector in LMH cell. (**d**) The protein expression of *IGF2BP1* after 48 h transfection of *IGF2BP1* overexpression vector in LMH cell. (**e**) The expression of genes related to fatter acid metabolism after transfection of si-IGF2BP1 in LMH cell. (**f**) The expression of genes related to fatter acid metabolism after transfection of pcDNA3.1-IGF2BP1 in LMH cell. The data are mean ± S.E.M. with four samples (*n* = 4/treatment group). Independent sample *t* test was used to analyze the statistical differences between groups. * *p* < 0.05; ** *p* < 0.01.

To study the function of *IGF2BP1* in chicken liver, we synthesized *IGF2BP1* specific siRNA and constructed *IGF2BP1* overexpression vector. Among three synthesized *IGF2BP1* specific siRNAs, only one of them can significantly inhibit *IGF2BP1* expression in LMH cell (Figure 4a,b), so we then used this siRNA to do the following study. For *IGF2BP1* overexpression, transfection of pcDNA3.1-IGF2BP1 plasmid can significantly induce *IGF2BP1* expression (Figure 4c,d). Because the liver is important for fatty acid metabolism, we then test the function of *IGF2BP1* on the expression of genes implicated in this process. Results showed that *IGF2BP1* inhibition in LMH can repress the expression of many genes related to fatty acid metabolism (Figure 4e), and the overexpression of *IGF2BP1* induced these genes expression (Figure 4f). Therefore, these results indicated that *IGF2BP1* promotes the expression of genes involved in fatty acid metabolism in LMH cell.

### 2.5. IGF2BP1 Promotes Chicken Adipocyte Proliferation

**Figure 5:**
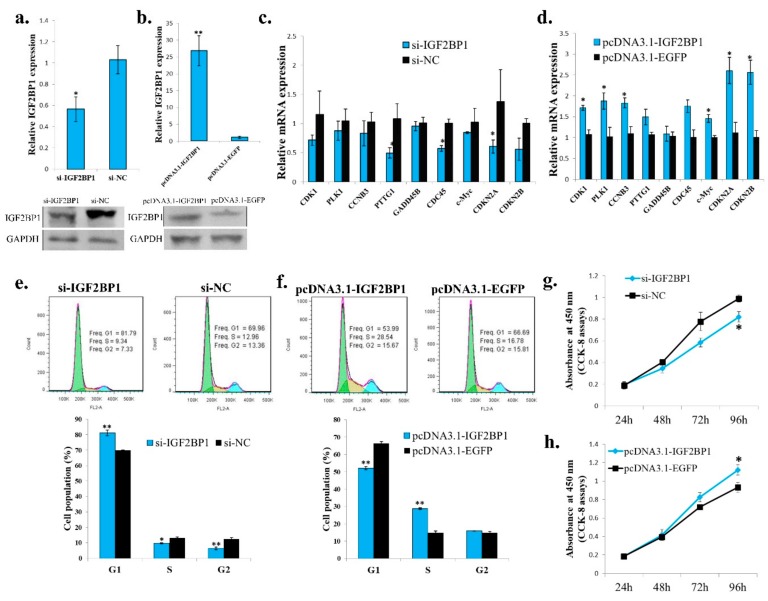
*IGF2BP1* promotes chicken adipocyte proliferation. (**a**) The mRNA and protein expression of *IGF2BP1* after 48 h transfection of si-IGF2BP1 in an ICP cell. (**b**) The mRNA and protein expression of *IGF2BP1* after 48 h transfection of *IGF2BP1* overexpression vector in an ICP cell. (**c**). The expression of cell cycle related genes after transfection of si-IGF2BP1 in an ICP cell. (**d**) The expression of cell cycle related genes after overexpression of *IGF2BP1* in an ICP cell. (**e**) *IGF2BP1* inhibition induced cell cycle arrest in an ICP cell. (**f**) *IGF2BP1* overexpression promote cell cycle progress in an ICP cell. (**g**) *IGF2BP1* inhibition repressed cell proliferation in an ICP cell. (**h**) *IGF2BP1* overexpression promote cell proliferation in an ICP cell. The data are mean ± S.E.M. with at least 3 samples (*n* ≥ 3/treatment group). Independent sample *t* test was used to analyze the statistical differences between groups. * *p* < 0.05; ** *p* < 0.01.

Because IGF2BP1 is an *IGF2* mRNA binding protein, and the IGF2 is an important growth factor, we then test the function of *IGF2BP1* in chicken adipocyte growth. The *IGF2BP1* specific siRNA can also inhibit *IGF2BP1* expression in an ICP cell (Figure 5a), and the *IGF2BP1* overexpression vector can promote *IGF2BP1* expression in an ICP cell (Figure 5b). *IGF2BP1* inhibition reduced the expression of genes involved in cell cycle pathway (Figure 5c), and the overexpression of *IGF2BP1* promoted the expression of these genes (Figure 5d). Additionally, *IGF2BP1* inhibition increased the cell populations arrest in the G1 phase but decreased the cell populations in the S phase (Figure 5e). *IGF2BP1* overexpression decreased the cell populations arrest in the G1 phase but increased the cell populations in the S phase (Figure 5f).These results suggesting that the *IGF2BP1* may promote cell proliferation by inducing cell cycle-related genes. Finally, CCK-8 assays also showed that *IGF2BP1* is able to increases cell number in an ICP cell (Figure 5g,h). Therefore, these results indicate that *IGF2BP1* promotes chicken adipocyte proliferation. 

### 2.6. IGF2BP1 Promotes Chicken Adipocyte Differentiation and Increases Lipid Droplet Accumulation

**Figure 6:**
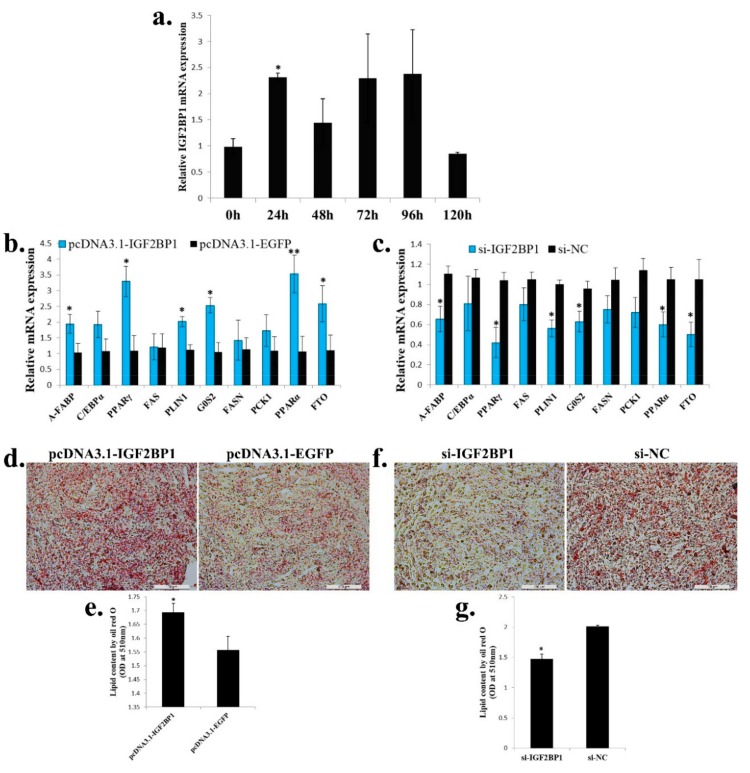
*IGF2BP1* promotes chicken adipocyte differentiation and increases lipid droplet accumulation. (**a**) The expression of *IGF2BP1* during ICP cell differentiation. (**b**) The expression of genes related to adipocyte differentiation and fatty acid metabolism after *IGF2BP1* overexpression in an ICP cell. (**c**). The expression of genes related to adipocyte differentiation and fatty acid metabolism after inhibition of *IGF2BP1* expression in an ICP cell. (**d**). Representative images of oil red O staining (red) after overexpression of *IGF2BP1* in an ICP cell; scale bar: 100 μm. (**e**). Lipid droplet content by oil red O staining and extraction method of cells transfected with *IGF2BP1* overexpression vector. (**f**). Representative images of oil red O staining (red) after inhibition of *IGF2BP1* in an ICP cell; scale bar: 100 μm. (**g**). Lipid droplet content by oil red O staining and extraction method of cells transfected with si-IGF2BP1 and si-NC. The data are mean ± S.E.M. with four samples (*n* = 4/treatment group). Independent sample *t* test was used to analyze the statistical differences between groups. * *p* < 0.05; ** *p* < 0.01.

Next, we test the function of *IGF2BP1* in adipogenesis. During ICP cell differentiation, *IGF2BP1* expression was immediately up-regulated after ICP cells were induced to differentiation at 24 h, but its expression was reverted to a low level at 120 h after differentiation (Figure 6a). Overexpression of *IGF2BP1* promoted adipocyte differentiation marker genes expression, and the genes related to fatty acid metabolism such as *PCK1*, *PPARα*, and *FTO* were also increased (Figure 6b). On the other hand, *IGF2BP1* inhibition repressed adipocyte differentiation marker genes expression, and the expression of genes which related to fatty acid metabolism were also decreased (Figure 6c). To investigated whether *IGF2BP1* can further affect lipid droplet accumulation during ICP differentiation, we transfected *IGF2BP1* overexpression vector or siRNA to the ICP and then induced the cells to differentiation for 48 h. As judged by oil red O staining and extraction assays, *IGF2BP1* overexpression significantly increased lipid droplet accumulation (Figure 6d,e), whereas *IGF2BP1* inhibition decreased lipid droplet content relative to control (Figure 6f,g). Taken together, these data indicate that *IGF2BP1* promotes chicken adipocyte differentiation and increases lipid droplet accumulation. 

## 3. Discussion

In this study, we used HFD to feed the Chinese native broilers and found that HFD can increase abdominal fat rate without affecting the body weight. Integrative analysis of the gene expression profiles of livers and abdominal fats between the HFD and ND chickens revealed that many DEGs are associated with lipid metabolic process and fat deposition pathways. Notably, the integrative analysis result indicated that *IGF2BP1* may be an important gene involved in chicken abdominal fat deposition. The expression of mane genes related to fatty acid metabolism were regulated by *IGF2BP1* in chicken liver cells, and the proliferation and differentiation of chicken adipocyte were also regulated by *IGF2BP1*. These results suggest that *IGF2PB1* is an important regulator for chicken abdominal fat deposition.

A HFD has a high content of energy and fat. A long-term high-fat diet would lead to excessive fat synthesis that cannot be absorbed and used by the tissues and organs. Therefore, it is well-known that high-fat diet can induce obesity, increase lipid accumulation in adipose tissues, and promote the infiltration of fat in other tissues and organs [15]. Excessive lipid accumulation in tissues may have a negative effect on human health, and the obesity would increase the likelihood of various diseases and conditions, such as type 2 diabetes, cardiovascular diseases, and depression [16,17]. In the broiler industry, the problem of excessive fat deposition in broilers is becoming more and more prominent with the constant improvement of the growth speed of broilers. The excessive fat deposition not only reduced the immunity of broilers to diseases, but also reduced the economic value of the broilers and produced waste that is hard to handle. Therefore, reducing broilers fat deposition, especially abdominal fat deposition, has become one of the main tasks for researchers in broiler industry. However, the mechanisms underlying the abdominal fat deposition of broilers still remain unclear. Even though it has been found that many factors were associated with chicken abdominal fat deposition [18], the detailed functions and mechanisms of these factors in abdominal fat deposition remain poorly understood. In this study, we aim to find some important genes involved in broiler abdominal fat deposition by analysis of DEGs between HFD and ND chickens, because the HFD would induce the expression of genes implicated in adipogenesis and fat deposition [19]. The liver and abdominal fat used as RNA-seq samples in this study were the most important organ and tissue for fat metabolism. The liver is the major site for converting excess carbohydrates and proteins into triglyceride and fatty acids, which are then exported and stored in adipose tissue. The abdominal fat is not only a storage site of fat but also a tissue associated with a clustering of metabolic risk factors as well as insulin resistance [20]. By analyzing the DEGs in liver and abdominal fat between the HFD and ND chickens, we found that a total of 146 genes were differentially expressed both in liver and in abdominal fat. Among these DEGs, *IGF2BP1* has attracted our attention because of it is a mRNA binding protein of *IGF2*, which has profound effects in organism development and metabolism [21]. Additionally, *IGF2BP1* is a gene that has never been study in chicken fat metabolism, and it is involved in the GH/IGF growth axis, which is critical for chicken growth. In many cases, the excessive abdominal fat deposition of the native chicken breeds accompanies the increase in growth rate. It would be interesting to study the correlation of growth acceleration and abdominal fat deposition in subsequent studies.

IGF2 is not only a crucial fetal growth factor but also is suggested to have metabolic effects postnatally [22]. *IGF2* overexpression in adult mice liver would decrease fat body mass [23], and low levels of circulating IGF2 concentrations were associated with weight gain and obesity in humans [24]. A paternally expressed quantitative trait locus (QTL) has a major effect on fat deposition maps to the *IGF2* locus in pigs [25]. IGF2BP1 can bind to the 5’UTR of *IGF2* mRNA and regulate *IGF2* translation. However, as an important *IGF2* mRNA binding protein, *IGF2BP1* has never been reported to be implicated with fat metabolism of fat deposition. In this study, we not only found that the expression of *IGF2BP1* was significantly induced in liver and abdominal fat of high-fat diet broilers, but also validated that *IGF2BP1* can regulate adipocyte proliferation and abdominal fat deposition. The function of *IGF2BP1* in fat metabolism of broilers may depend on its binding and regulation on *IGF2*. It has been found that high-fat diet can induce *IGF2* mRNA expression in sprague dawley rat offspring [26], but in our results the *IGF2* mRNA was never been induce by high-fat diet. The high-fat diet may induce IGF2 protein expression by promoting *IGF2BP1* expression.

In many cases, a high-fat diet can promote abdominal fat deposition and increase body weight. However, in our study, we found that high-fat diet induced broilers abdominal fat deposition without significantly affect their body weight. One potential reason underlying this phenomenon is that the broilers used in this study were sex-linked dwarf chickens with a missense mutation in *GHR* exon 5. GHR is a receptor of growth hormones, which play an important role in the regulation of animal growth [27]. In chickens, several kinds of GHR mutations would result in dwarfism and reduced growth. These GHR mutations were called *dw* genes. These *dw* genes can reduce or abolish the binding affinity of GH to the receptor, interrupt the signal transduction of GH-GHR-IGFs, and influence the normal development of chickens [28,29,30]. The interruption of GH-GHR-IGFs signaling in *dw* chickens might reduce the effect of high-fat diet on body weight. However, the function of *dw* gene in fat deposition still work in process. Because a high-fat diet significantly increased the abdominal fat deposition of these *dw* chickens. 

It has been showed that *GHR* knockout would result in dwarfism, reduced body weight and obesity [31], and many SNPs in *GHR* gene were associated with fat deposition in human and chickens [32,33]. In China, a great number of chicken breeds contained a sex-linked mutation in the *GHR* gene, which is called *dw* gene as mention above. Chickens with *dw* gene have many advantages in chicken production, such as less feed consumption, higher feed conversion efficiency, smaller occupancy of chicken house, good egg-laying performance, and satisfactory meat quality [34]. Therefore, the *dw* gene is widely used in China chicken breeding. However, more and more chicken farms reflected that the chickens with *dw* gene have more abdominal fat deposition than the chickens without. This phenomenon is similar to the results of *GHR* gene knockout in mice, which showed signs of obesity [31,35]. The detailed mechanism underlying how *GHR* gene mutation or knockout result in obesity and abdominal fat deposition still remain unclear, especially in chickens. It is well-known that the expression of many genes and non-coding RNAs related to lipid metabolism and fat deposition would be significantly changed during high-fat diet-induced obesity [36,37]. In this study, we used high-fat diet to identify key genes and pathways associated with abdominal fat deposition in *dw* chickens, and we found that *IGF2BP1* is one of the candidate gene affecting *dw* chicken abdominal fat deposition. *IGF2BP1* not only can regulate genes participate in fat metabolism, but also the genes related to adipogenesis, which is a key process in fat mass increase, were be influenced. However, the involvement of *IGF2BP1* seems modest in some of our results, especially for lipid droplet accumulation. One of the potential reasons may be that our experimental methods are not comprehensive enough. Both overexpression plasmid and siRNA method belong to transient transfection. The adipogenesis and lipid droplet accumulation are processes that need a long time induction. The use of lentiviral infection or gene knock-out method may see a more significant effect of *IGF2BP1* on adipogenesis. Additionally, the function of *IGF2BP1* is mainly depends on the regulation of *IGF2* mRNA translation. But it has showed that *IGF2* has more expression on liver, kidney, and heart [38]. The function of *IGF2* on abdominal fat or ICP cell may be limited, as well as the function of *IGF2BP1*. However, all of these statements are limited to speculation. The detailed function of IGF2BP1 in chicken adipogenesis, the mechanism of how *IGF2BP1* increased its expression in a high-fat diet-fed *dw* chickens, the connection between *IGF2BP1* expression and GHR-mutation, and the specific pathways involved in GHR-mutation induced obesity of chicken still remain to be further explored.

## 4. Experimental Section

### 4.1. Animals, Tissue Collection and RNA sequencing

A total of 12 eight-week-old female dwarf type Xinghua (XH) chickens with similar body weight (with *growth hormone receptor* gene exon 5 a T/C mutation, this type of chicken has better meat flavor and is very popular in South China chicken market, but it has excessive abdominal fat deposition) were reared in individual cages and divided into two groups (*n* = 6 in each group). One group of chickens were fed a high-fat diet (HFD) consisting of 40% carbohydrate, 25% fat, and 20% protein for 2 weeks, the other group chickens were fed a normal diet (ND, control group) consisting of 41% carbohydrate, 5% fat, and 22% protein for 2 weeks. Then the chickens were slaughtered, and the weight of eviscerated carcasses and abdominal fat were be recorded to calculate the abdominal fat weight and abdominal fat rate. The tissues of abdominal fat and liver were rapidly collected, immediately frozen in liquid nitrogen and stored at −80 °C until use. Abdominal fats and livers collected from three HFD chickens, which have higher abdominal fat rate than the other three HFD chickens, were selected for RNA sequencing. Similarly, abdominal fats and livers collected from three ND chickens, which have lower abdominal fat rate than the other three ND chickens, were selected for RNA sequencing. The tissue samples were sent to Beijing Genomics Institute for RNA sequencing. Total RNA for RNA-seq was isolated from samples using Trizol Reagent (Invitrogen, Carlsbad, CA, USA) according to the manufacturer’s protocol. RNA quantity and quality were evaluated on an Agilent 2100 Bioanalyzer (Agilent Technologies, Waldbronn, Germany), and RNA integrity was further examined using agarose gel electrophoresis. High-throughput RNA-seq was performed on the BGISEQ-500 platform (BGI, Wuhan, China). |log2FC| ≥ 0.5, *p* ≤ 0.001 was set as the threshold for selection of differentially expressed gene. All the sequence data have been deposited in NCBI’s Gene Expression Omnibus (GEO, http://www.ncbi.nlm.nih.gov/geo) and are accessible through GEO series accession number GSE129840. 

### 4.2. Histology

H-E staining of the chicken abdominal fat was carried out as previously described [39]. Briefly, after fixation with 4% paraformaldehyde, the abdominal fat samples were embedded in paraffin, and 12-μm-thick serial sections were made. The sections were then stained with H-E stain following standard protocols. Microscopic observation and photograph were taken with Leica DM2500 microscope (Leica, Wetzlar, Germany). Adipocytes area and diameter were calculated using Nikon Eclipse Ti microscope and NIS-Elements BR software (Nikon, Tokyo, Japan).

### 4.3. Cell Culture and Transfection

Immortalized chicken preadipocytes (ICP) was a kind gift of the Poultry Breeding Group of the College of Animal Science and Technology, Northeast Agricultural University. Chicken Liver Hepatocellular carcinoma cell line (LMH) and ICP were cultured in high-glucose Dulbecco’s modified Eagle’s medium (Gibco, Carlsbad, CA, USA) with 10% foetal bovine serum and 0.2% penicillin/streptomycin, at 37 °C with 5% CO_2_. To induce ICP differentiation, we added 160 μM sodium oleate (Sigma Life Science, St. Louis, MO, USA) to the medium [2]. Plasmid DNA and siRNA transfection were carried out using the transfection reagent Lipofectamine 3000 (Invitrogen Corporation, Carlsbad, CA, USA) following the manufacturer’s protocol.

### 4.4. RNA Extraction, cDNA Synthesis and Quantitative Real-Time PCR

Total RNA was extracted from tissues or cells using RNAiso reagent (Takara, Otsu, Japan). The reverse transcription reaction for mRNA was performed with PrimeScript RT reagent Kit with gDNA Eraser (Takara) according to manufacturer’s manual. qPCR program was carried out in Bio-rad CFX96 Real-Time Detection System (Bio-rad, Hercules, CA, USA) with iTaq™ Universal SYBR® Green Supermix (Bio-rad). All reactions were run in triplicate. The 2^−ΔΔ*C*t^ method was used to measure gene expression with *β-actin* (For liver tissues and LMH cells) or *GAPDH* (For abdominal fats and ICP cells) as the reference gene.

### 4.5. Plasmid Construction and siRNA

*IGF2BP1* coding sequence were amplified from chicken embryonic cDNA by PCR using gene-specific clone primers (Supplementary File 4). The PCR product was cloned into the pcDNA3.1 vector (Invitrogen). The successful *IGF2BP1* overexpression vector was confirmed by agarose gel electrophoresis and DNA sequencing. siRNA specifically against chicken *IGF2BP1* was obtained from RiboBio, and a nonspecific duplex was used as the control.

### 4.6. Western Blotting

Western blotting was performed according to the standard procedures. The primary antibodies used in this study were showed as following: anti-IGF2BP1 (Santa Cruz Biotechnology, Santa Cruz, CA, USA), anti-GAPDH (Bioworld, St Louis Park, MN, USA). 

### 4.7. Cell Cycle Analysis

After 48 h transfection of gene overexpression vectors or siRNA, cells were collected and fixed in 75% ethanol overnight at −20 °C. After ethanol fixation, the cells were stained with propidium iodide using PI/RNase staining buffer (BD Biosciences Pharmingen, San Diego, CA, USA) according to the manufacturer’s manual. BD Accuri C6 flow cytometer (BD Biosciences, San Diego, CA, USA) was subsequently used to analyze the cell cycle, and the data analysis was performed using FlowJo 7.6 software (TreeStar Inc., Ashland, OR, USA).

### 4.8. Oil Red O Staining and Quantification

After transfected with *IGF2BP1* overexpression vector or siRNA, ICP cells were washed with PBS and fixed in 4% formaldehyde for 10 min. Then the cells were stained with Oil-Red-O working solution (Solarbio, Beijing, China) according to the manufacturer’s manual. After washed with ddH2O, the cells were analyzed using a microscope (Leica). The Oil-Red-O dyes were then extracted in isopropanol solution containing 4% Nonidet P-40 and quantified by NanoDrop 2000C spectrophotometers (Thermo Fisher Scientific, San Jose, CA, USA) at 510 nm.

### 4.9. CCK-8 Assay

ICP cells that transfected with *IGF2BP1* overexpression vector or siRNA were cultured in 96-well plates. After 24 h of transfection, 10 μL of cell counting kit-8 reagent (Dojindo, Kumamoto, Japan) was added into each well and incubated for 1 h. The absorbance was measured at 450 nm by a Model 680 Microplate Reader (Bio-Rad). The assay was repeated at different time points of 24, 48, 72, 96 h after transfection. All the data were acquired by averaging the results from three independent experiments.

### 4.10. Statistical Analysis

Data are presented as mean ± S.E.M of at least three replicates. Differences between groups were assessed using one sample *t*-test. *p* < 0.05 was considered statistically significant. All experiments were carried out at least three times.

### 4.11. Bioinformatics Analysis

Gene ontology (GO) analysis of the enriched genes was performed using the web-based Metascape (a gene annotation & analysis resource, http://metascape.org/gp/index.html#/main/step1). The Venn diagram was calculated and draw by a web-based software (http://bioinformatics.psb.ugent.be/webtools/Venn/).

### 4.12. Ethics Standards

Animal experiments were performed in accordance with the regulations and guidelines established by the Animal Care Committee of South China Agricultural University (approval number: SCAU#0017; 21 November 2017). 

## 5. Conclusions

In conclusion, our study revealed that a high-fat diet would increase abdominal fat deposition and induce adipocyte hypertrophy in broiler. By analyzed DEGs between high-fat diet and normal diet broilers in liver and abdominal fat, we found that the DEGs are involved in some pathways related to fat metabolism, such as PPAR signaling, fat digestion and absorption, ECM-receptor interaction, and steroid hormone biosynthesis. Notably, we found that *IGF2BP1* is an important gene that can be induced by a high-fat diet in liver and abdominal fat of broilers. *IGF2BP1* can regulate the expression of genes related to fatty acid metabolism, promote adipocyte proliferation and differentiation, and increase lipid droplet accumulation. This study provides new insights into understanding the genes and pathways involved in abdominal fat deposition of broiler and found that *IGF2BP1* is an important regulator for chicken adipogenesis.

## Supplementary Materials

Supplementary materials can be found at https://www.mdpi.com/1422-0067/20/12/2923/s1.
