# Supplementary material for: Integrative Analyses of mRNA Expression Profile Reveal the Involvement of IGF2BP1 in Chicken Adipogenesis
Source: Int J Mol Sci. 2019 Jun 14;20(12):2923. doi: 10.3390/ijms20122923 (PMC6627201; doi:10.3390/ijms20122923)
Supplement: Supplementary file 1 [file ijms-20-02923-s001.zip › ijms-499619-supplementary/supplementary file 4.docx]

Integrative analyses of mRNA expression profile reveal the involvement of *IGF2BP1* in chicken abdominal fat deposition

Jiahui Chen^1,2,3,#^, Xueyi Ren^1,2,3,#^, Limin Li^1,2,3^, Shiyi Lu^1,2,3^, Tian Chen^1,2,3^, Liangtian Tan^1,2,3^, Manqing Liu^1,2,3^, Qingbin Luo^1,2,3^, Shaodong Liang^1,2,3^, Qinghua Nie^1,2,3^, Xiquan Zhang^1,2,3^, Wen Luo^1,2,3,*^

**For cloning the following primers were used for PCR:**

IGF2BP1 CDS (reference sequence: NM_205071.1) cloning for pcDNA3.1 overexpression vector:

s: 5’- CGGGATCCGCCACCATGCCGCTCAGCGCCAGCCTCCC

as: 5’- CGGAATTCAAGTATAAATCCAGTTCCCAC

**For quantitative real time RT-PCR, the following primers were used:**

c-Myc-qPCR (reference sequence: KU981087):

s: 5’- GCCAGCGAAGGAATGAGC

as: 5’- CGTCCGATTGGATAGACAGAAC

PLK1-qPCR (reference sequence: NM_001030639.1):

s: 5’- AACTGAACGGGCGAAAG

as: 5’- GGGAATACAGGCTGGGTC

CDK1-qPCR (reference sequence: NM_205314.1):

s: 5’- AAGTGAGGAGGAAGGTG

as: 5’- AATGGCAGAAGACGATA

CCNB3-qPCR (reference sequence: NM_205239.2):

s: 5’- TCCTCATCGCCTCCAAA

as: 5’- CAGGGTCATCTCACAGACAAAA

PTTG1-qPCR (reference sequence: XM_025154831.1):

s: 5’- GAAAGGCTCTTGGAAA

as: 5’- TGAGGAGGTCTCGTTATT

GADD45B-qPCR (reference sequence: XM_015299957.2):

s: 5’- GCTTCGGTCCCTTTGGTGA

as: 5’- CCGGCAGTTGTTGTGCAGTC

CDC45-qPCR (reference sequence: XM_415070.6):

s: 5’- TGTCTCGCCATAACCA

as: 5’- GCCGCTTCTGTCCTT

CDKN2A-qPCR (reference sequence: NM_204434.1):

s: 5’- TACGTTCTCCCTTCACCTCC

as: 5’- AAACCGCTTCAACTGACTACAT

CDKN2B-qPCR (reference sequence: NM_204433.1):

s: 5’- AAACCCACCCGCTCTTCC

as: 5’- CCGTCCCTTTCGGCTTCA

β-actin-qPCR (reference sequence: NM_205518.1):

s: 5’- TTGTTGACAATGGCTCCGGT

as: 5’- AACCATCACACCCTGATGTCT

IGF2BP1-qPCR (reference sequence: NM_205071.1):

s: 5’- AAGGCACAAGGCAGGATT

as: 5’- GCAGCTCATTGACGGTTTT

A-FABP-qPCR (reference sequence: FJ493543.1):

s: 5’- AGTTTGTGGGCACCTGGAAGC

as: 5’- CCATCCACCACTTTCCTCTT

C/EBPα-qPCR (reference sequence: NM_001031459.1):

s: 5’- GGAGCAAGCCAACTTCTACGC

as: 5’- CTCGTTCTCGCAGATGTCGC

PPARγ-qPCR (reference sequence: NM_001001460.1):

s: 5’- TACATAAAGTCCTTCCCGCTGACC

as: 5’- TCCAGTGCGTTGAACTTCACAGC

FAS-qPCR (reference sequence: NM_001199487.1):

s: 5’- AAGGAGGAAGTCAACGG

as: 5’- TTGATGGTGAGGAGTCG

PLIN1-qPCR (reference sequence: NM_001127439.1):

s: 5’- ATGGAAGGGCCAAGGAGAAC

as: 5’- CCCGACATGCCAAAGTGTTG

G0S2-qPCR (reference sequence: NM_001190924.3):

s: 5’- CGGGGCGAAAGAGCTGAG

as: 5’- AGCACGTACAGCTTCACCAT

FASN-qPCR (reference sequence: NM_205155.3):

s: 5’- GCTAAGATGGCATTGCACGG

as: 5’- TGCCAGAGCCTCCACTATCT

PCK1-qPCR (reference sequence: NM_205471.1):

s: 5’- CCAGATAATGGGGAGCCGTG

as: 5’- CAGGTCTGCGACCTCCAAAT

PPARα-qPCR (reference sequence: NM_001001464.1):

s: 5’- AGTAAGCTCTCAGAAACTTTGTTG

as: 5’- GTCATTTCACTTCACGCAGCA

FTO-qPCR (reference sequence: NM_001185147.1):

s: 5’- TTTTGGTTTCAAGGCGAGCG

as: 5’- CCAGGCTCGTCATAAGCTCC

ACSL5-qPCR (reference sequence: NM_001031237.1):

s: 5’- CCCTAAAGGTGCCATGCTGA

as: 5’- CTCCGCAGCTGTACATCACA

CPT1A-qPCR (reference sequence: NM_001012898.1):

s: 5’- ACAGCGAATGAAAGCAGGGT

as: 5’- GCCATGGCTAAGGTTTTCGT

CYP7A1-qPCR (reference sequence: NM_001001753.1):

s: 5’- GTAACGCCCTAGATGCCCTC

as: 5’- GCTCTCTCTGTTTCCCGCTT

CYP8B1-qPCR (reference sequence: NM_001005571.1):

s: 5’- CATCATTCCCTGGCTGGGTT

as: 5’- TAGCCAAAAACCCGGAGGAC

CIDEC-qPCR (reference sequence: NM_001277678.1):

s: 5’- GGTGTAGGCTCAGTTCCGT

as: 5’- TATTTGGAGACAGGCGCAGC

GAPDH-qPCR (reference sequence: NM_204305.1):

s: 5’- AGAACATCATCCCAGCGT

as: 5’- AGCCTTCACTACCCTCTTG

PCK1-qPCR (reference sequence: NM_205471.1):

s: 5’- CAACACCAGATTCCCAGGCT

as: 5’- AAAGGAGATCCAATCGGCCC

OPN2SW-qPCR (reference sequence: NM_205517.2):

s: 5’- CTCATGAACAAGCAGTTCCGC

as: 5’- GTCGTCCCCGAACGGACT

ERNI-qPCR (reference sequence: NM_001080874.1):

s: 5’- AACGGGGGAGGCTTATCTCT

as: 5’- ATCAGTCTCCACTGTTGCGG

ELF3-qPCR (reference sequence: XM_419257.6):

s: 5’- CACAGACAAGCCTGAGTGGT

as: 5’- CGGTCATACAGCTCATCCCC

CYP4B1-qPCR (reference sequence: NM_001329509.1):

s: 5’- GTAGCCCTCATGGCAGAGTC

as: 5’- GCTCAAAGAGCTCCACCGAT
